# The comparison of long-term oncological outcomes and complications after proximal gastrectomy with double tract reconstruction versus total gastrectomy for proximal gastric cancer

**DOI:** 10.1186/s12957-023-02985-z

**Published:** 2023-03-23

**Authors:** Keming Ying, Weisong Bai, Guiru Yan, Ziseng Xu, Shenheng Du, Chengxue Dang

**Affiliations:** 1 Department of Surgical Oncology, The First Affiliated Hospital of Xi’an Jiaotong University, No. 277, West Yanta Road, Xi’an, 710061 Shaanxi Province China; 2 Department of Surgical Oncology, The Central Hospital of Hanzhong, Hanzhong, Shaanxi Province, China

**Keywords:** Proximal gastrectomy; Double tract reconstruction; Total gastrectomy; Survival; Postoperative complications

## Abstract

**Background:**

Conventional methods for treating patients with proximal gastric cancer (PGC) include proximal gastrectomy (PG) and total gastrectomy (TG) and such methods have become challenging due to double tract reconstruction (DTR). However, the clinical outcomes remain unclear. This study was performed with the aim of verifying that PG-DTR was beneficial in terms of reducing the incidence of postoperative complications and improving the prognosis.

**Methods:**

The PGC patient cohort was retrospectively grouped into the PG-DTR and TG groups. Clinicopathological features, complications, and survival data were compared between the two groups.

**Results:**

A total of 388 patients were included in the analyses. Patients who were subjected to TG tended to have more severe gastroesophageal reflux (GR) (*P* = 0.041), anemia (*P* = 0.007), and hypoalbuminemia (*P* < 0.001). Overall survival rates, regardless of clinical stage, were significantly different between the PG-DTR and TG groups (all *P* < 0.05). The multivariate Cox regression analysis confirmed that surgical procedure, tumor size, infiltration depth, lymph node metastasis, differentiation, and age were independent risk factors. The patients were likely to benefit from PG-DTR (all HR > 1 and* P* < 0.05). However, no significant differences were observed in the risks of GR, anemia, and hypoalbuminemia (all *P* > 0.05). Moreover, the nomogram derived from significant parameters showed great calibration and discrimination ability and significant clinical benefit.

**Conclusions:**

The patients who underwent PG-DTR had a favorable prognosis. The risk of postoperative complications, such as severe GR, anemia, and hypoalbuminemia, was lower in PG-DTR than in TG. Thus, PG-DTR is more beneficial for patients with PGC and may be a valuable and promising surgical procedure.

## Background

More than one million new cases of gastric cancer and 800,000 deaths from gastric cancer are reported every year. Gastric cancer is the fifth most frequently diagnosed cancer and the third leading cause of cancer death worldwide [1]. The incidence of gastric cancer has been declining worldwide, but the incidence of proximal gastric cancer (PGC) has been increasing in recent decades [2–4]. According to the fifth edition of the Japanese guidelines for the treatment of gastric cancer (2018) [5], proximal gastrectomy (PG) is recommended for patients with early upper gastric cancer who can retain more than one half of the distal gastric stump after R0 resection. In PG, patients are able to retain normal digestive function of the residual stomach in which digestion and absorption of food is promoted to the greatest extent; the procedure has the advantages of a simple anastomosis method, a small resection area, and a low risk of injury. However, due to gastroesophageal reflux (GR) and other symptoms, patients who undergo PG often have difficulty eating postoperatively, a poor nutritional status, and a poor quality of life, thus leading to low immunity and may have an increased risk of tumor recurrence or metastasis. In addition, residual lymph nodes in some areas may lead to incomplete treatment, which is a mental stress or for the patient.

Total gastrectomy (TG) is a common surgical treatment method for PGC, and its advantages are as follows. First, we can remove the possible metastatic lymph nodes around the distal residual stomach, patients can avoid severe GR after PG. Therefore, Western scholars believe that TG has better clinical efficacy than PG [6]. Our previous study also demonstrated [7] that TG not only alleviated the symptoms of GR but also improved the survival rate of patients and was superior to PG for PGC. Although TG significantly relieved the symptoms of GR, TG was reported to cause “postgastrectomy syndrome,” which leads to a significant decrease in the patient’s food intake, and ultimately, patients suffer from severe nutritional metabolism-related complications, such as anemia, hypoproteinemia, and thinness, which are also obvious after surgery.

In both PG and TG, the defects and complications after digestive tract reconstruction are very obvious, the quality of life of the patients is affected to varying degrees, and the postoperative survival time of the patients is shortened. Therefore, to reduce the occurrence of the above situations and improve the postoperative efficacy of treatment for patients with PGC, we must explore, effectively implement, and optimize digestive tract reconstruction for PGC. Moreover, Li et al. [8] reported that it is urgent to promote the “Chinese consensus on digestive tract reconstruction after proximal gastrectomy” to guide and optimize PGC treatment in the future.

To prevent GR after surgery for PGC, Japanese scholars first reported jejunal interposition reconstruction [9]. However, this type of surgery was still not particularly effective in relieving GR symptoms. Subsequently, Aikou et al. [10], a Japanese scholar, reported the application of interposition jejunum with double tract reconstruction (DTR) for the reconstruction of the gastrointestinal tract after PG. DTR has been shown to be suitable for most gastrointestinal reconstructions after resection of proximal gastric cancer [9]. This surgical method does not have high requirements for retaining residual gastric volume and is especially suitable for patients with excessive gastric resection and unsuitable for esophagogastric anastomosis. In addition, this surgical method also has a good antireflux effect. The study results reported by Wang et al. [11] showed that compared with TG, PG-DTR could increase total protein, albumin, and hemoglobin levels in plasma after surgery. Since the short-term efficacy of PG-DTR in patients with PGC is satisfactory, we wondered if the use of PG-DTR could improve the long-term therapeutic effect in postoperative patients and likely avoid total gastric resection in patients with PGC. However, it has not yet been determined whether PG-DTR improves survival outcomes or reduces the risk of postoperative complications in patients with PGC. Moreover, it is unknown whether PG-DTR can improve long-term oncologic outcomes in patients. Therefore, we hypothesized that PG-DTR might be beneficial for improving survival time and reducing the risk of postoperative complications in patients with PGC. To test this hypothesis, we analyzed and compared the short-term and long-term oncologic outcomes following PG-DTR versus TG for PGC in this patient population.

## Methods

**Fig. 1:**
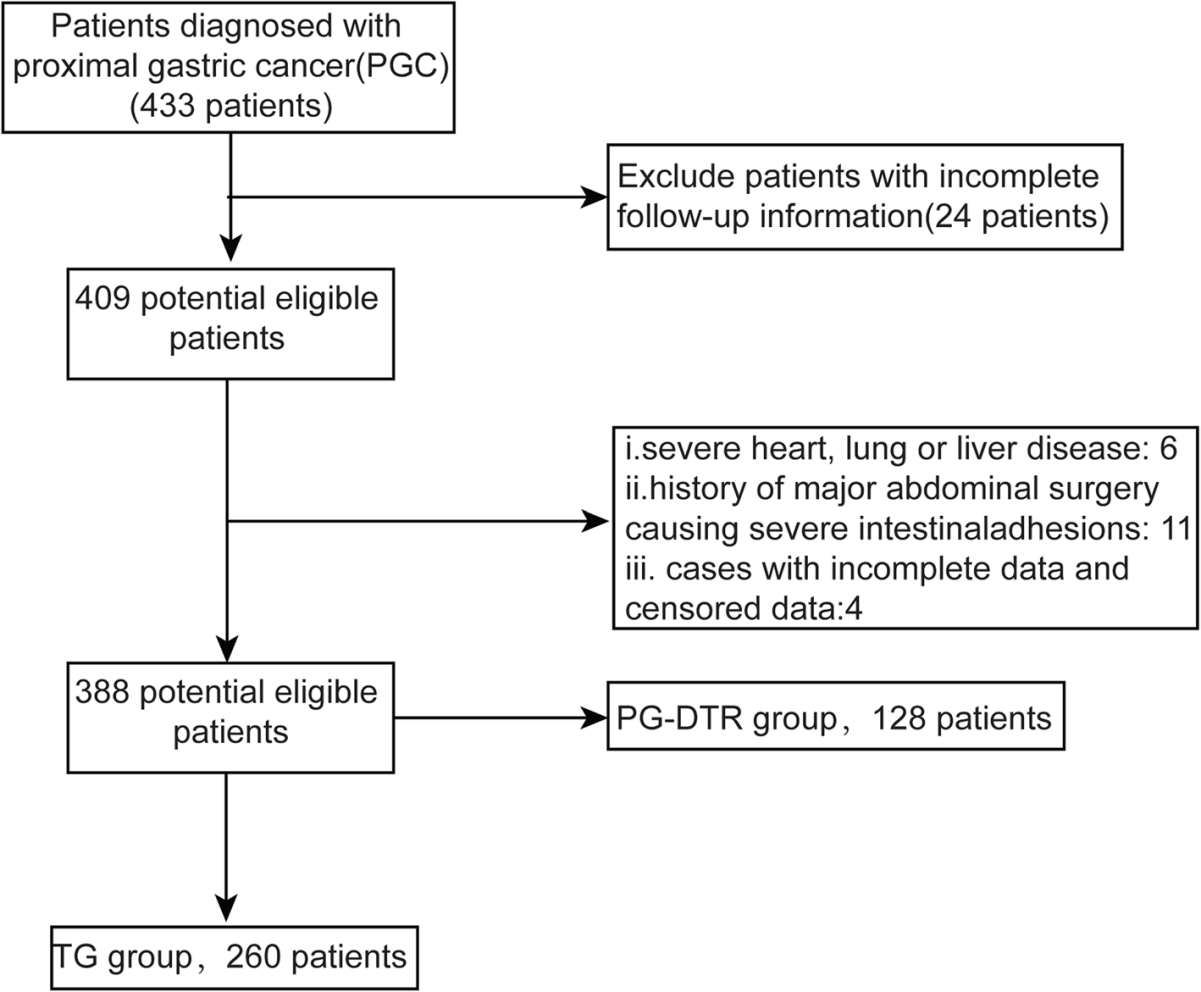
Patient inclusion criteria

We retrospectively analyzed 388 consecutive PGC patients who underwent radical gastrectomy at the Central Hospital of Hanzhong and the First Affiliated Hospital of Xi’an Jiaotong University between April 2013 and June 2020. The surgery indication was selected based on the location of the tumor. Inclusion criteria: patients were diagnosed with PGC by electronic gastroscopy and pathological examination before surgery, patients with no surgical contraindications, and patients suitable for R_0_ resection. Exclusion criteria: patients with severe heart, lung, or liver disease, patients with a history of major abdominal surgery causing severe intestinal adhesions, patients with previous or concurrent malignancies of other sites, patients who underwent preoperative neoadjuvant chemotherapy, patients who underwent emergency surgery or with acute pyloric obstruction, patients reporting postoperative alcohol use, and patients with a smoking history. The patients were divided into two groups: the PG-DTR group (128 patients) and the TG group (260 patients) (Fig. 1).

During the study period, patients who underwent PG-DTR or TG underwent the same preoperative workup and surgery preparations. All procedures were directed and performed by specialists who strictly followed the principles of radical resection of PGC in the Department of Surgical Oncology. Postoperative management and policies were similar in both surgical approaches. Thirty-eight patients were lost to follow-up, and 152 patients died before having a primary end-point event. The median follow-up time was 50.3 months.

### Surgical procedure

#### Proximal gastrectomy with double tract reconstruction

**Fig. 2:**
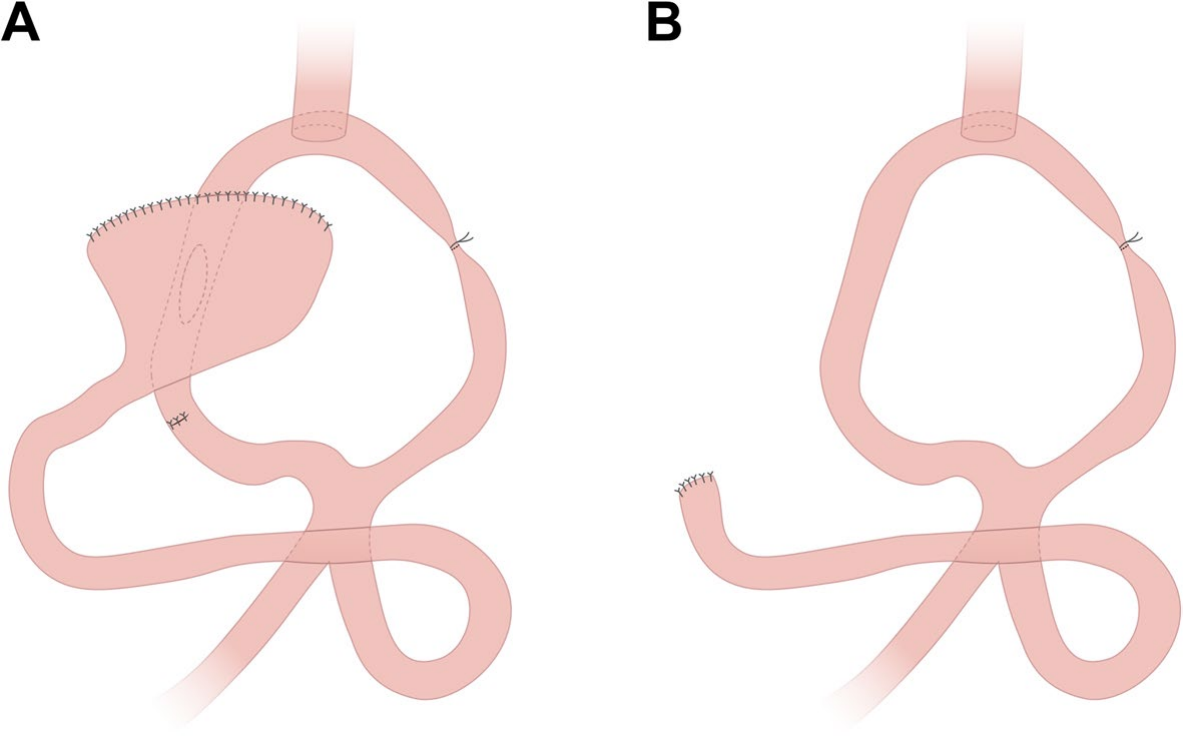
**A** Schematic representation of proximal gastrectomy with double tract reconstruction. **B** Schematic representation of total gastrectomy

PG-DTR was performed via transabdominal radical resection of the proximal stomach and standard D2 regional lymphadenectomy. Approximately 20 ~ 25 cm from the Treitz ligament, the side of the jejunum was lifted in front of the colon and anastomosed with the end of the esophagus using a tube stapler. Side-to-side anastomosis of the jejunum output loop and the posterior wall of the remnant stomach was performed approximately 35 cm away from the anastomotic site using another tube stapler. Braun anastomosis was performed between the output loop and the input loop 10 cm from the gastrojejunostomy and approximately 5 cm from the ligament of Treitz. Finally, the jejunum was sutured to the Braun side by applying 2–3 stitches to narrow one half of the intestinal tube, which played a regulating role. Some of the food passes through the jejunum into the stomach, and the remaining portion of the food directly passes through the jejunum. The afferent loop on the other side was closed and blocked with no. 7 silk thread approximately 5–7 cm from the esophagojejunostomy site. Digestive tract reconstruction was accomplished, and all anastomotic stomas were reinforced with no. 1 surgical sutures (Fig. 2A).

#### Total gastrectomy

TG was performed by transabdominal radical resection of the total stomach and standard D2 regional lymphadenectomy. Based on the location and growth pattern of the tumor, the esophagus was cut off at an appropriate distance above the cardia, the anvil of the stapler was inserted into the esophageal stump, and the esophageal stump was closed using purse-string suture. The duodenal stump was closed with a cutting stapler and the specimen was removed. The jejunal loop was raised approximately 35 cm below the ligament of Treitz, and the central rod was threaded through the bowel wall to engage the esophageal stump anvil. Jejunoesophageal anastomosis was completed. The left input loop was blocked by ligation 5 to 7 cm from the esophagojejunal anastomosis. Finally, Braun anastomosis was performed between the jejunum loop and the efferent loop 20 cm from the esophagojejunal anastomotic ostium. Digestive tract reconstruction was accomplished, and all anastomotic stomas were reinforced with no. 1 surgical sutures (Fig. 2B).

### Classification and grading

A total of 388 PGC patients who underwent PG-DTR or TG suffered from postoperative GR, which was graded using the DeMeester method [12]. According to the severity of heartburn and acid reflux (HAR) symptoms, GR was classified as grade 0 to III. Grade 0 was asymptomatic, grade I presented occasional HAR but required no treatment, grade II presented frequent HAR that required treatment, and grade III patients suffered from frequent and severe HAR that affected their normal life.

Postoperative patients with occasional or asymptomatic (grade 0 or I) HAR were stratified into the no GR group, whereas patients with frequent and severe HAR (grade II or grade III) were added to the GR group.

To control the influencing factors, acid suppression drugs were prohibited in the no GR group patients during the study period. After confirming the patients in the GR group, we appropriately administered acid suppression drugs to these patients to relieve frequent and severe postoperative HAR.

### Definition

Anemia was defined as a hemoglobin level of less than 110 g/L, hypoalbuminemia was defined as a serum albumin level of less than 35 g/L, and postgastrectomy syndrome is a series of symptoms and signs that present due to complications of gastrectomy that affect the quality of life of gastric cancer patients postoperatively. Severe heart disease is defined as heart disease in which patients cannot tolerate surgery due to symptoms such as severe decline in cardiac function and arrhythmia. Severe liver disease is defined as liver disease in which patients cannot tolerate surgery due to symptoms such as severe decline in liver function, jaundice, hypoproteinemia, and ascites. In distant metastasis, gastric cancer cells invade tissues and organs other than the stomach, such as the liver, lung, brain, bone, and distant lymph nodes, and form new metastatic lesions at metastatic sites. Local recurrence is defined as local recurrence of gastric cancer after surgery or even anastomotic recurrence.

### Statistical analysis

The patients’ clinicopathological features, preoperative situation, operative details, postoperative outcomes, postoperative complications, and follow-up status were retrospectively collected and entered into a PGC database. On the grounds of the patient’s intention to participate, the analysis was performed in the postoperative follow-up between-group comparisons.

The Mann‒Whitney *U* test was used to compare two groups of continuous variables. Comparisons of the ordinal data or categorical data were processed by the chi-squared test or Fisher’s exact test. Continuous variables are expressed as interquartile ranges and median values. The survival rate was analyzed using the Kaplan‒Meier method, and comparisons of variables were processed using the log-rank test. Multivariate analysis was performed using Cox regression analysis variables that were statistically significant (*P* < 0.05) in the univariate analysis.

The final predictive nomogram was estimated by the C-index, calibration curve, and time-dependent receiver operating characteristic curve (ROC). Clinical usage was evaluated by decision curve analysis (DCA).

All statistical analyses were performed with SPSS software (version 23.0, SPSS Inc., Chicago, IL) and RStudio (version 4.0; https://www.rstudio.com/). Statistical significance levels were determined by a two-sided test, and *P* < 0.05 was defined as statistically significant.

## Results

### Patients’ clinicopathologic features and complications

**Fig. 3:**
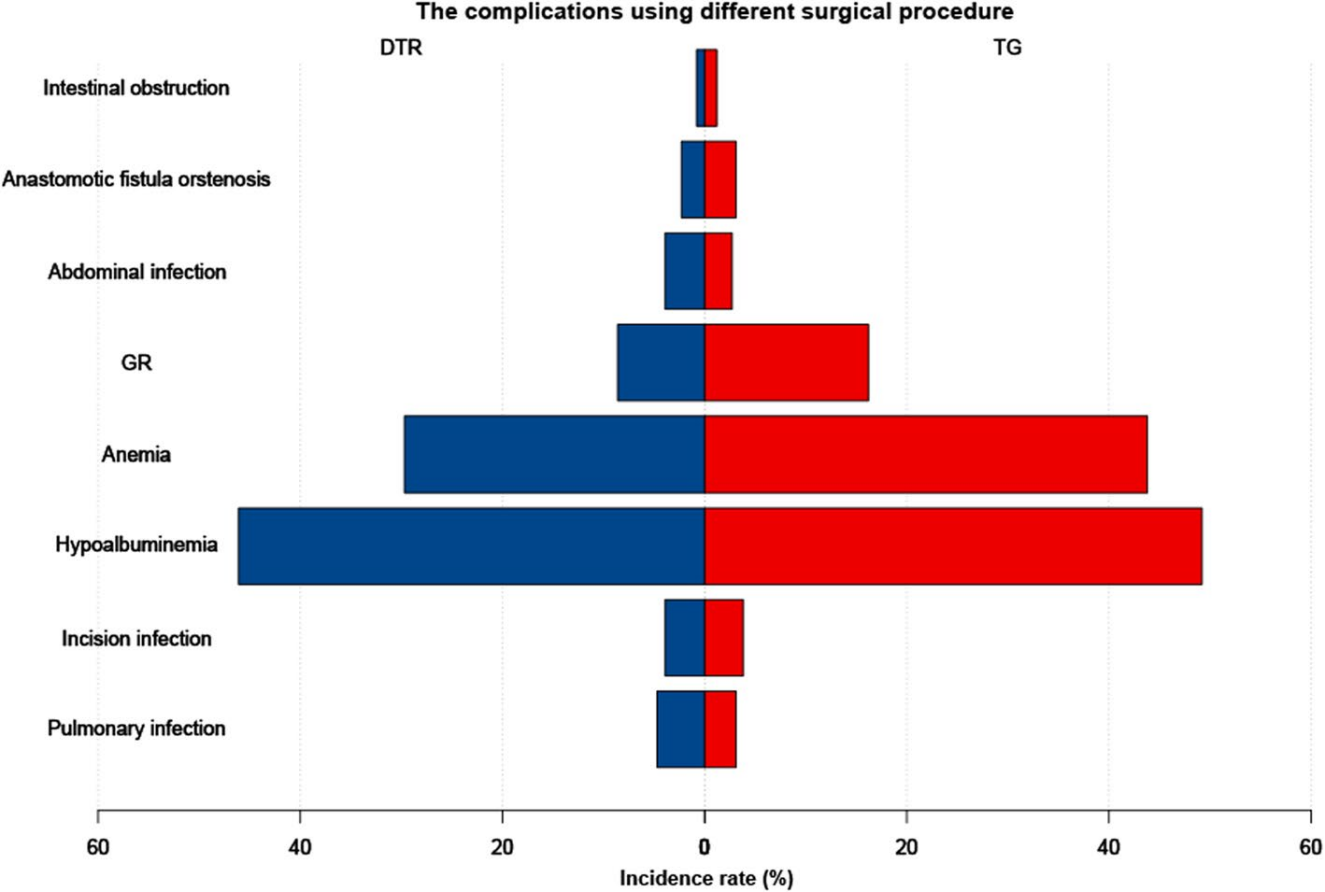
Comparison of the incidence of postoperative complications between the PG-DTR group and the TG group. PG proximal gastrectomy, DTR double tract reconstruction, TG total gastrectomy, GR gastroesophageal reflux. Incision infection, pulmonary infection, abdominal infection, anastomotic fistula or stenosis, and intestinal obstruction were the most commonly observed short-term complications observed at the 3-month postoperative follow-up. GR, anemia, and hypoproteinemia were considered to be long-term complications and indicated nutritional status at the 1-year postoperative follow-up

The baseline clinical and pathological characteristics of the PGC patients are summarized in Table 1. The results demonstrated that the baseline clinical parameters and pathological indexes showed no significant difference, which indicated that patients who underwent PG-DTR or TG were homogeneous and comparable (all *P* > 0.05, Table 1). Additionally, the postoperative complications in the PG-DTR group and the TG group are also presented in Table 2. There were no significant differences in the risk of early postoperative complications regardless of infectious complications or others between the PG-DTR group and the TG group (all *P* > 0.05), and the incidence of adverse events during hospitalization in the patients who underwent PG-DTR was not higher than that in the patients who underwent TG. Moreover, the incidence of long-term nutritional complications, such as GR (8.6% vs. 16.2%, *P* = 0.041, Fig. 3 and Table 2), anemia (29.7% vs. 43.8%, *P* = 0.007, Fig. 3 and Table 2), and hypoalbuminemia (21.1% vs. 49.2%,* P* < 0.001, Fig. 3 and Table 2), at 1 year postoperatively remarkably declined in the PG-DTR group. The results showed that PGC patients who underwent TG were more likely to have long-term postoperative malnutrition. Therefore, PG-DTR could reduce the risk of long-term postoperative complications in PGC patients.

**Table 1:** Comparison of clinicopathological features in the PG-DTR and TG groups

	PG-DTR (*n* = 128)	TG (*n* = 260)	*P* value
Gender			0.467
Male	41 (32.0%)	93 (35.8%)	
Female	87 (68.0%)	167 (64.2%)	
Age (years)	62 (53–68)	64.5 (56–71)	0.053
Tumor size (cm)			0.883
≥ 5	66 (51.6%)	132 (50.8%)	
< 5	62 (48.4%)	128 (49.2%)	
Differentiation			0.733
Well	44 (34.8%)	97 (37.3%)	
Moderate	48 (37.7%)	99 (38.1%)	
Poor	36 (27.5%)	64 (24.6%)	
Preoperative GR			0.153
Yes	5 (3.9%)	20 (7.7%)	
No	123 (96.1%)		
Preoperative anemia		240 (92.3%)	0.940
Yes	12 (9.4%)	25 (9.6%)	
No	116 (90.6%)	235 (90.4%)	
Preoperative hypoalbuminemia			0.556
Yes	11 (8.6%)	18 (6.9%)	
No	117 (91.4%)	242 (93.1%)	
Pathological type			0.425
Papillary adenocarcinoma	51 (39.8%)	103 (39.6%)	
Canalicular adenoma	45 (35.2%)	95 (36.5%)	
Mucinous adenocarcinoma	13 (10.1%)	26 (10.0%)	
Signet ring carcinoma	12 (9.4%)	13 (5.1%)	
Other	7 (5.5%)	23 (8.8%)	
Infiltration depth			0.857
T1	81 (63.3%)	155 (59.6%)	
T2	28 (21.9%)	58 (22.3%)	
T3	7 (5.5%)	16 (6.2%)	
T4	12 (9.3)	31 (11.9%)	
Total operative time (min)	280 (190–385)	275 (205–378)	0.578
Blood loss (ml)	150 (58–183)	148 (77–195)	0.089
Lymph node metastasis			0.946
N0	60 (46.9%)	123 (47.3%)	
N1	42 (32.8%)	79 (30.3%)	
N2	21 (16.4%)	48 (18.5%)	
N3	5 (3.9%)	10 (3.9%)	
Pathological TNM stage			0.883
I	53 (41.4%)	105 (40.4%)	
II	55 (43.0%)	118 (45.4%)	
III	20 (15.6%)	37 (14.2%)	
Vessel carcinoma embolus			0.514
Yes	59 (46.1%)	129 (49.6%)	
No	69 (53.9%)	131 (50.4%)	
Nerve invasion			0.192
Yes	55 (42.9%)	130 (50.0%)	
No	73 (57.1%)	130 (50.0%)	
Distant metastasis			0.394
Yes	58 (45.3%)	106 (40.8%)	
No	70 (54.7%)	154 (51.2%)	
Local recurrence			0.921
Yes	52 (40.6%)	107 (41.2%)	
No	76 (59.4%)	153 (58.8%)	

The *P* value of measurement data was obtained from the Mann–Whitney *U* test, and the *P* value of categorical data was obtained from the chi-squared test

*PG* proximal gastrectomy, *DTR* double tract reconstruction, *TG* total gastrectomy

**Table 2:** Comparison of postoperative complications in the PG-DTR and TG groups

	PG-DTR (*n* = 128)	TG (*n* = 260)	*P* value
Incision infection			0.977
Yes	5 (3.9%)	10 (3.8%)	
No	123 (96.1%)	250 (96.2%)	
Pulmonary infection			0.424
Yes	6 (4.7%)	8 (3.1%)	
No	122 (95.3%)	252 (96.9%)	
Abdominal infection			0.516
Yes	5 (3.9%)	7 (2.7%)	
No	123 (96.1%)	253 (97.3%)	
Anastomotic fistula or stenosis			0.682
Yes	3 (2.3%)	8 (3.1%)	
No	125 (97.7%)	252 (96.9%)	
Intestinal obstruction			0.733
Yes	1 (0.8%)	3 (1.2%)	
No	127 (99.2%)	257 (98.8%)	
GR			0.041
Yes	11 (8.6%)	42 (16.2%)	
No	117 (91.4%)	218 (83.8%)	
Anemia			0.007
Yes	38 (29.7%)	114 (43.8%)	
No	90 (70.3%)	146 (56.2%)	
Hypoalbuminemia			< 0.001
Yes	27 (21.1%)	128 (49.2%)	
No	101 (78.9%)	132 (50.8%)	

*PG* proximal gastrectomy, *DTR* double tract reconstruction, *TG* total gastrectomy, *GR* gastroesophageal reflux. GR, anemia, and hypoproteinemia were the indicators of nutritional follow-up 1 year after the operation. The *P* value of categorical data was obtained from the chi-squared test

### The prognostic validation of the PG-DTR surgical procedure

**Fig. 4:**
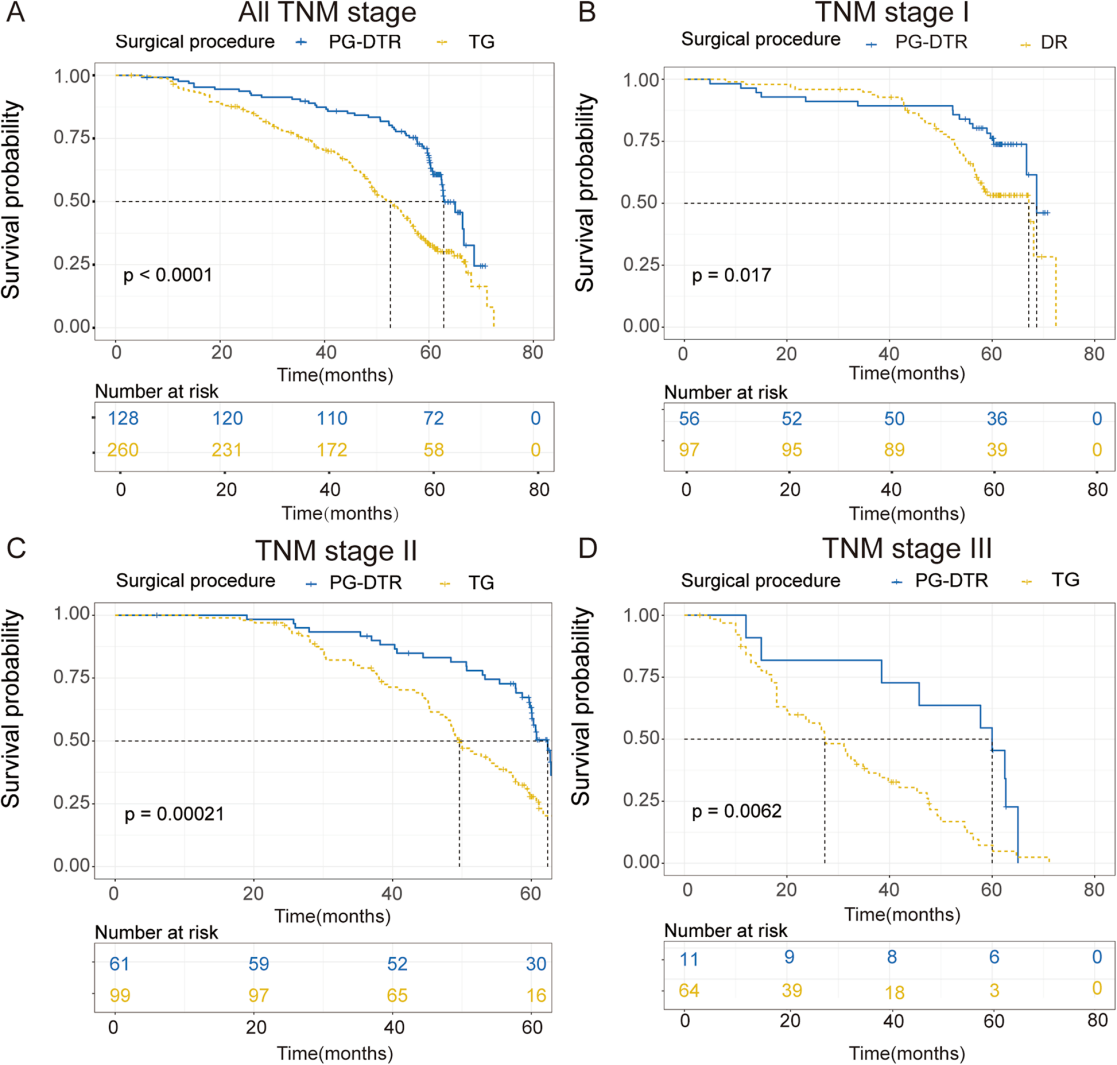
Kaplan‒Meier analyses of the PG-DTR group and the TG group. **A** Kaplan‒Meier analysis of all PGC patients. **B** Kaplan‒Meier analysis of stage I PGC patients. **C** Kaplan‒Meier analysis of stage II PGC patients. **D** Kaplan‒Meier analysis of stage III PGC patients. PG proximal gastrectomy, DTR double tract reconstruction, TG total gastrectomy. The *P* value was obtained from the log-rank test

Kaplan‒Meier analysis was performed to investigate whether the prognosis of PG was improved after PG-DTR. As shown in Fig. 4A, the prognosis of patients who underwent PG-DTR was significantly prolonged (*P* < 0.001), and the results verified that the PG-DTR surgical procedure not only decreased malnutrition complications but also improved the prognosis for PGC patients. In addition, we further explored the prognosis of patients in various clinical stages to decrease the influence of confounding factors based on the stratification analysis. As expected, the survival time of PGC patients was remarkably prolonged after undergoing the PG-DTR surgical procedure in various subgroups (Fig. 4B–D, all *P* < 0.05). Consequently, we demonstrated that PG-DTR could be beneficial for increasing the survival time of PGC patients.

### Establishment and assessment of the comprehensive nomogram

**Fig. 5:**
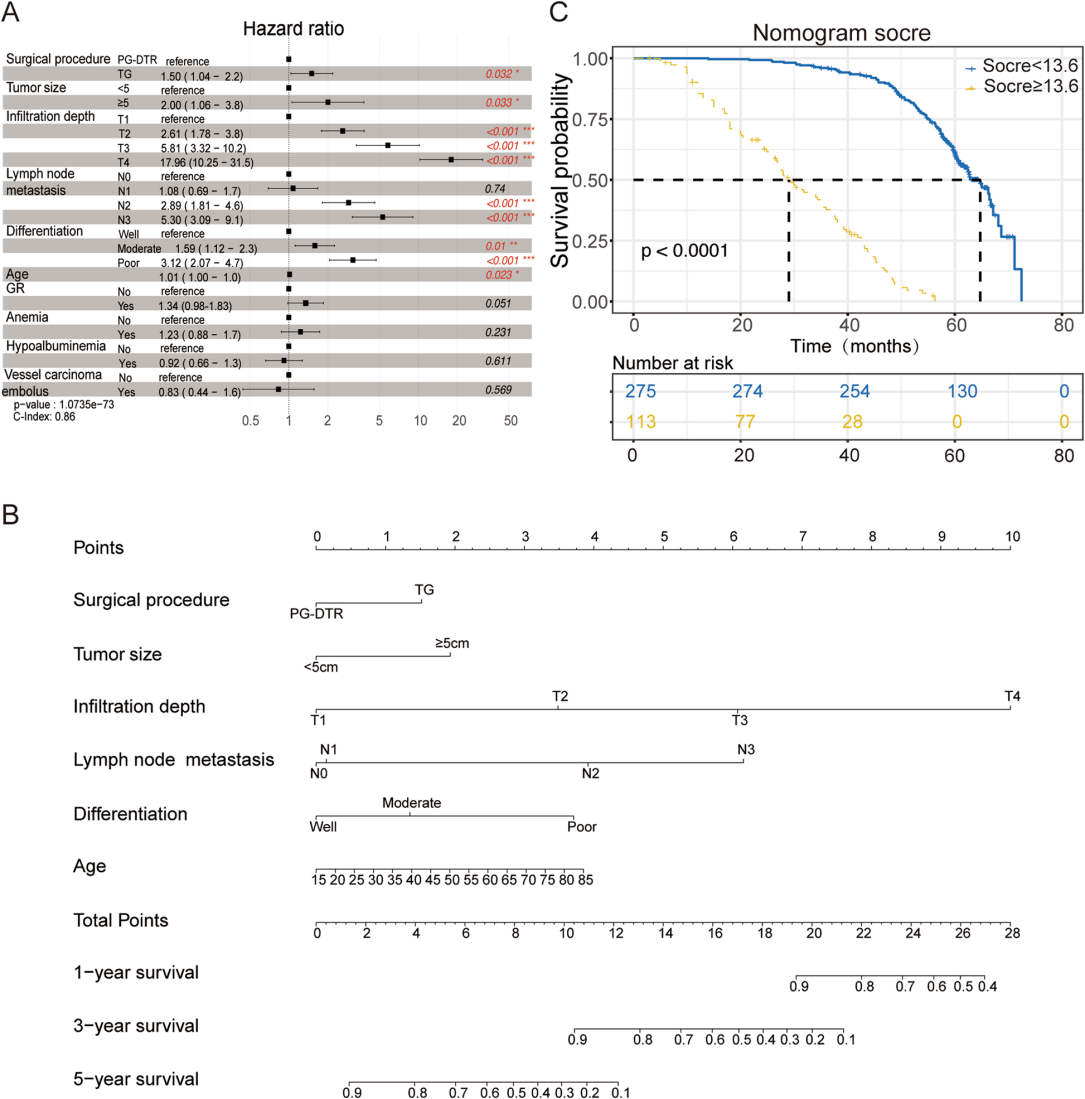
Cox regression analysis and development of a nomogram in PGC patients. **A** Forest plot showing the results of the multivariate Cox regression analysis. **B** A comprehensive nomogram was established to predict the 1-, 3-, and 5-year survival probabilities in PGC patients. **C** Kaplan‒Meier analysis of the nomogram and the cutoff value obtained from X-tile. The *P* values were obtained from the Cox regression and Kaplan‒Meier analysis

**Fig. 6:**
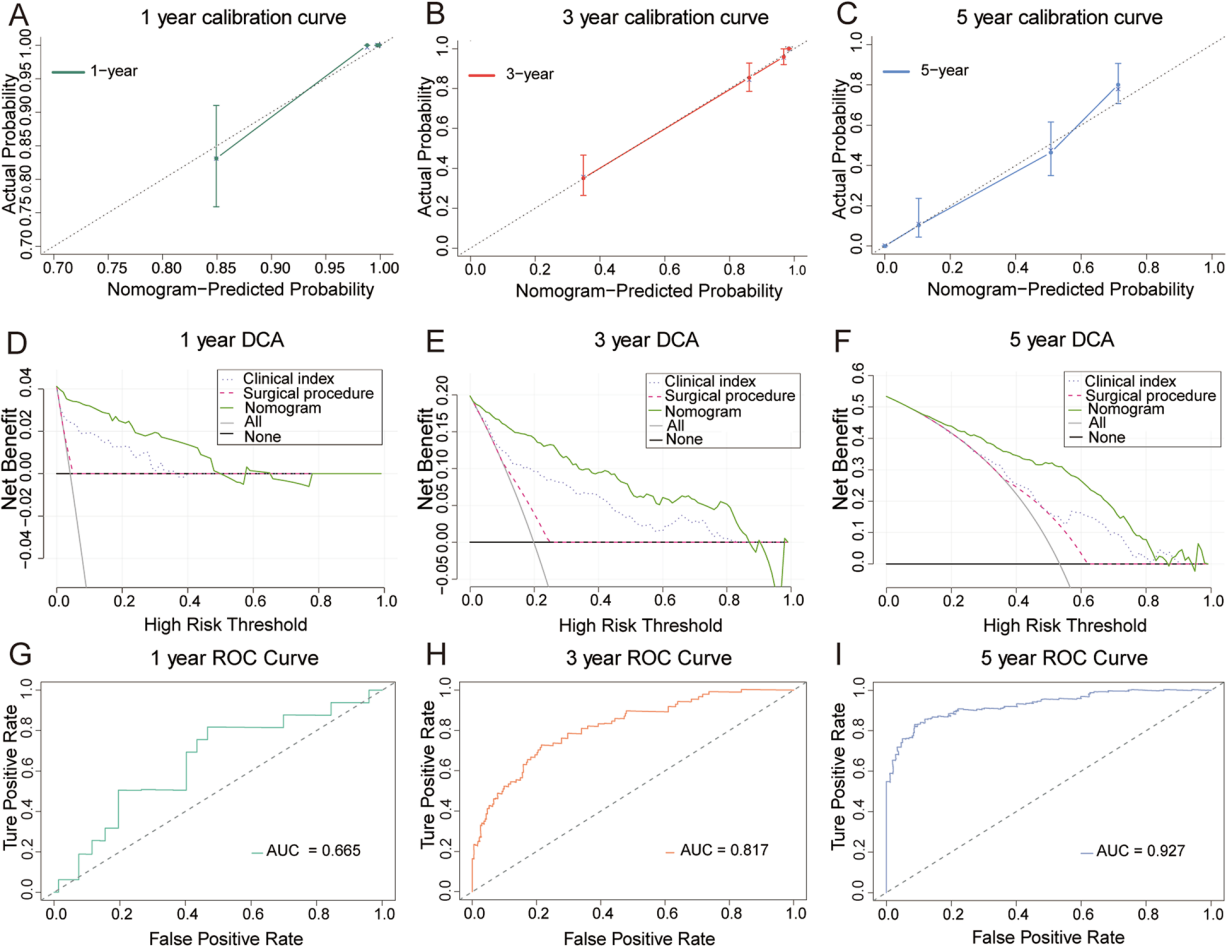
The performance of the nomogram. **A-C** The calibration curves of 1-, 3-, and 5-year showed more appropriate calibration ability in PGC patients, in which the blue dotted lines represent the ideal predictive model, and the red solid line represents the nomogram model. **D-F** Time-dependent ROC curve analysis for the nomogram of the 1-, 3-, and 5-year survival of PGC patients. **G-I** The DCA curves showed a comparable net benefit if the threshold probability for a patient or a doctor was within a range from 0 to 0.80 during 1, 3, and 5 years. The *y*-axis represents the net benefit. The *x*-axis represents the predicted overall survival probability. The oblique smooth solid line represents a kind of hypothesis that all patients survive in the corresponding time. The horizontal smooth solid line represents the hypothesis that none of the patients survive for more than 1 year. **G-I** Time-dependent ROC curve analysis for the nomogram at 1, 3, and 5 years in PGC patients

According to our previous results, we found the advantages of PG-DTR in terms of improving clinical outcomes and complications. Therefore, we evaluated the prognostic value of the PG-DTR method combined with the underlying clinical indicators to establish a more accurate nomogram for the postoperative assessment of PGC patient survival. Thus, univariate Cox regression followed by multivariate Cox regression was utilized to identify reliable independent risk or protective factors in PGC patients. The results showed that surgical procedure, tumor size, infiltration depth, lymph node metastasis, differentiation, and age presented independent risk factors based on Cox regression analysis, and the patients were likely to benefit from treatment with the PG-DTR surgical method (all HR > 1 and* P* < 0.05, Table 3 and Fig. 5A). However, the complications of GR, anemia, and hypoalbuminemia did not present statistically significant differences, which suggested that these indexes could not be predictive indicators. Therefore, considering all the previously mentioned significant predictive parameters, we developed a comprehensive nomogram. As shown in Fig. 5B, the survival probability of PGC patients at 1, 3, and 5 years could be estimated by using this nomogram, which was derived from the sum of each parameter score. We also calculated the uncorrected and corrected C-index, which were 0.866 and 0.854, respectively. Moreover, a higher risk score was related to a more serious prognosis according to the Kaplan‒Meier analysis (Fig. 5C). Of note, X-tile was performed to distinguish the cutoff value. To evaluate the performance of the nomogram, calibration curves and time-dependent ROC analysis were used to assess the calibration and discrimination ability in PGC patients. Moreover, DCA was performed to evaluate the clinical usage of the nomogram. The results verified suitable calibration at 1, 3, and 5 years (Fig. 6A–C). The decision curves showed that if the threshold probability was between 0 and 0.80, then using the comprehensive nomogram to predict prognosis added more benefit than treating either all or no patients, which was more reliable than the signal surgical method or clinicopathological indictors (Fig. 6D–F). The time-dependent ROC also presented satisfying AUC values at 1, 3, and 5 years, which were 0.665, 0.817 and 0.927, respectively (Fig. 6G–I). These results indicated that the nomogram could improve current treatment standards for PGC patients.

**Table 3:** Univariate and multivariate Cox analysis of variables with overall survival

Variables	Univariate Cox regression		Multivariate Cox regression	
	HR (95% CI)	*P* value	HR (95% CI)	*P* value
Surgical procedure		< 0.001		0.032
PG-DTR	1 (reference)		1 (reference)	
TG	2.25 (1.66–3.06)		1.50 (1.04–2.18)	
Tumor size (cm)		< 0.001		0.033
< 5	1 (reference)		1 (reference)	
≥ 5	1.99(1.52–2.61)		2.01 (1.06–3.80)	
GR		0.021		0.051
No	1 (reference)		1 (reference)	
Yes	1.38 (1.05–1.82)		1.34 (0.98–1.83)	
Infiltration depth		< 0.001		< 0.001
T1	1 (reference)		1 (reference)	
T2	4.51 (3.26–6.22)		2.61 (1.78–3.82)	
T3	9.10 (5.51–15.03)		5.81 (3.32–10.16)	
T4	49.59 (30.76–79.93)		17.96 (10.25–31.47)	
Lymph node metastasis		< 0.001		< 0.001
N0	1 (reference)		1 (reference)	
N1	1.32 (0.87–2.02)		1.08 (0.69–1.68)	
N2	5.69 (3.76–8.60)		2.89 (1.81–4.61)	
N3	17.81 (11.38–27.86)		5.30 (3.09–9.10)	
Differentiation		< 0.001		< 0.001
Well	1 (reference)		1 (reference)	
Moderate	2.12 (1.53–2.93)		1.59 (1.12–2.26)	
Poor	5.78 (4.04–8.26)		3.12 (2.07–4.72)	
Anemia		< 0.001		0.231
No	1 (reference)		1 (reference)	
Yes	2.76 (2.07–3.68)		1.23 (0.88–1.73)	
Hypoalbuminemia		< 0.001		0.611
No	1 (reference)		1 (reference)	
Yes	2.39 (1.83–3.12)		0.92 (0.66–1.28)	
Age (years)	1.03 (1.02–1.04)	< 0.001	1.01 (1.00–1.03)	0.023
Gender		0.337	–	
Female	1 (reference)			
Male	1.15 (0.87–1.51)			
Vessel carcinoma embolus		< 0.001		0.569
No	1 (reference)		1 (reference)	
Yes	1.73 (1.32–2.27)		0.83 (0.44–1.57)	
Nerve invasion		0.473	–	
No	1 (reference)			
Yes	1.10 (0.85–1.43)			

The *P* value was obtained from the Cox regression analysis

*PG* proximal gastrectomy, *DTR* double tract reconstruction, *TG* total gastrectomy, *GR* gastroesophageal reflux, *HR* hazard ratio, *CI* confidence interval

## Discussion

In this study, we found that PG-DTR was significantly higher than TG with respect to long-term oncological outcomes and prevention postoperative complications in patients with PGC. To clarify the effect of PG-DTR on patients with PGC, we performed a control study to compare the two surgical methods. The results were consistent in several sensitivity analyses and subgroup analyses. Collectively, our data demonstrated that PG-DTR is associated with a more favorable long-term survival and has advantages in reducing the risk of postoperative complications when compared with TG.

As shown in Fig. 4, we found that the overall survival of patients with PGC who were treated with PG-DTR was significantly better than that of patients treated with TG regardless of clinical stage. To evaluate the prognosis of PGC patients, the surgical procedure and relevant significance were integrated, and a comprehensive nomogram was established. The nomogram presented an appropriate discrimination and calibration ability, which indicated that these enrolled parameters could predict the prognosis of PGC patients (Figs. 5 and 6). As shown in Table 2, in terms of postoperative complications, PG-DTR was associated with a significantly lower incidence of postoperative anemia and hypoalbuminemia as well as fewer patients who developed postoperative GR. Considering the two components of the primary outcome, the long-term survival of patients with PGC and the cumulative incidence of postoperative complications were higher than those in the TG group. Perspectives may vary among surgeons and patients about which component poses a more worrisome issue, but complications are primarily associated with physical distress and a short survival time, which can have profound psychological implications for patients with PGC.

The long-term survival of patients with PGC in this study was as anticipated, and the PG-DTR group was similar to or even better than the TG group in previous studies. Fan et al. [13] compared PG-DTR (51 cases) and total gastrectomy with Roux-en-Y reconstruction (TG-RY) (81 cases) in the treatment of PGC and observed that the 3- and 5-year overall survival rates were 65.3 and 55.0% in the PG-DTR group and 63.8 and 47.2% in the TG-RY group, respectively. Although the difference in survival rate was not statistically significant, the authors still believed that PG-DTR was safe and feasible. Ji et al.’s [14] study showed that DTR was safe and reliable and did not affect the overall 3-year survival rate of patients. Ma et al. [15]reported that for individuals with pathological stage II and III PGC, the long-term survival rate associated with PG-DTR was potentially higher than that associated with TG. Therefore, to help determine the appropriate surgical approach and strategy for patients, the authors recommend using the results of their research as a guide for surgeons. A meta-analysis showed that compared with TG-RY, there was no significant difference in the 1-, 3-, and 5-year survival of PG-DTR, but the survival trend was still significant. In addition, in our study, PG-DTR was compared with TG, which was performed by Brownian anastomosis [16]. Based on propensity score matching analysis by Ko et al. [17], the authors reported that the 5-year overall survival rates of the PG-DTR group were significantly higher than those of the TG group (100% vs. 81.6%, respectively, *P* = 0.02). Consequently, the authors demonstrated that PG-DTR, which may be suitable for PGC, was associated with better clinical outcomes and survival. Our study showed that the 5-year overall survival rates of PGC patients after PG-DTR were higher than those of patients after TG (*P* < 0.01), which implied that after PG-DTR, patients have a relatively good prognosis and lower recurrence rate. The 5-year survival of the PG-DTR group was higher than that of the TG group regardless of the clinical stage (all *P* < 0.05). Therefore, our study results are similar to those of other reports.

There are several reasons why patients with PGC who undergo PG-DTR survive for a longer period. First, to date, there are many different types of surgical methods for postoperative reconstruction of PGC, and new surgical methods are still emerging, but satisfactory curative effects and survival cannot be achieved [9]. The ideal method of digestive tract reconstruction should meet the following requirements: (1) the procedure keeps food moving smoothly through the duodenum. (2) It can play the role of gastric bag storage and ensure good digestive tract absorption. (3) Patients are able to maintain a good nutritional status and a good quality of life after surgery. (4) The operation is safe and simple and has a low mortality risk. (5) The short-term and long-term oncologic outcomes were good or improved. In our study, it was clear that most surgical procedures, including Braun anastomosis of the esophagus and jejunum after TG, did not fully meet all of these requirements. However, the PG-DTR can basically meet the above reconstruction requirements in our experimental group. This may be the main reason why the survival rate of the patients in the PG-DTR group was higher than that in the TG group in our study.

The results of our study proved that compared with TG, PG-DTR not only has more favorable long-term survival but also has obvious advantages in alleviating postoperative complications such as severe GR. In addition, we speculated that there was some correlation between postoperative complications caused by different surgical procedures and the postoperative survival rate in patients with PGC.

The incidence of postoperative GR symptoms in traditional surgery of PGC was as high as 60% with minimal efficacy, of which 30% seriously affected the basic quality of life of patients [18]. Xu et al. [19] reported through another systematic review and meta-analysis that esophagojejunostomy with DTR reduce the incidences of GR and anastomotic stenosis. One study showed that DTR could better prevent reflux esophagitis and improve quality of life for patients who underwent radical PG, and GR had a linear relationship with the global health status score [14]. A systematic review found that DTR, which is a promising surgical method for patients with oesofagocardial gastric cancer, was associated with few complications related to GR disease and dysphagia [20]. Furthermore, a network meta-analysis indicated that compared with other surgical methods, PG-DTR can significantly reduce the incidence of postoperative reflux esophagitis and anastomotic stenosis by searching for articles published between January 1, 1990, and February 1, 2021 [21].

It should be noted that in the PG-DTR group, the residual stomach and esophagus were connected through the jejunum, and the jejunum could still have intestinal peristalsis, effectively resisting the upward reflux of the residual gastric digestive fluid. In addition, the double output channel can make the food shunt and reduce the residual stomach pressure and the occurrence of GR. Therefore, PG-DTR surgery could relieve the symptoms of acid reflux, heartburn, and postoperative GR in patients with PGC resection. Although multiple-factor regression analysis showed that postoperative GR was not significantly related to surgical procedures, the univariate-factor regression analysis showed that compared with TG, PG-DTR was statistically significantly associated with postoperative GR (*P* < 0.01). In addition, our previous studies have shown that severe postoperative GR in patients with PGC is likely to lead to recurrence, metastasis, and even reduced survival time [7]. Consequently, the lower incidence of postoperative GR may be the second most important reason for better survival with surgical PG-DTR. Therefore, it is important to select the proper PGC surgical procedure, such as PG-DTR, to prevent severe postoperative GR and improve the overall survival of patients.

To thoroughly remove the tumor as much as possible and clean the metastatic lymph nodes around the distal stomach, TG is currently the most commonly used surgical method for the treatment of PGC [9]. However, a series of nutritional metabolic syndromes, especially pernicious anemia and hypoproteinemia, are often unavoidable after TG. In China, Wang et al. [11] believed that compared with TG, PG-DTR could significantly improve the total protein, albumin, and hemoglobin levels in the plasma of patients after surgery. Jung et al. [22] reported that the safety of PG-DTR in the treatment of upper gastric cancer was similar to that of TG-RY, but patients with PG-DTR had higher hemoglobin and vitamin B levels in the long term. A meta-analysis showed that [23] compared with TG patients, PG-DTR patients had a better nutritional status (*P* < 0.05) and a higher postoperative vitamin B12 (*P* < 0.01) level. Another meta-analysis showed that PG-DTR can be recommended for application to upper-third EGC considering its superior postoperative nutritional outcome [24].

A systematic review and meta-analysis [25] found that compared with the TG group, the levels of nutritional indicators (vitamin B12 supplements and deficiency) were significantly higher in the PG-DTR group. This may be the cause of pernicious anemia in PGC patients who underwent TG. Cho et al. [26] suggested that the cumulative incidence of anemia and vitamin B12 deficiency was similar between the PG-DTR and TG groups. The reason for the lack of statistical significance is that the sample size of the above study was too small, comprising only 80 cases, while the sample size of our study was 388 cases. Du et al. [16] implied that compared with TG-RY, PG-DTR was associated with higher levels of postoperative 1-year albumin (OR = 1.90, 95% CI: 1.08 to 2.77, *P* < 0.001) and postoperative 1-year hemoglobin (OR = 5.07, 95% CI: 2.83 to 7.31, *P* < 0.001). In our study, anemia and hypoproteinemia were significant only in the univariate analysis, but there was no significant statistical significance in the multivariate analysis. The possible reason for this is that PG-DTR was compared with TG, which was performed by Brownian anastomosis instead of TG-RY. Hence, our results are basically consistent with the above studies.

In our study, we found that the mechanism of PG-DTR aligned with the mechanism of radical resection for PGC to a greater extent, ensuring a negative resection margin while preserving as much residual stomach as possible. Compared with TG, PG-DTR possesses the advantages of minimal trauma and a low risk of postoperative complications. This operation, which is beneficial for intestinal digestive absorption, improves the regulation of gastrointestinal hormones and promotes the absorption of iron, folic acid, vitamin 12, protein nutrients and trace elements, preserves the normal physiological channel of the human body, and makes digesta enter the duodenum to mix with digestive enzymes. As a result, postoperative anemia and hypoalbuminemia rates are low. In contrast, patients who suffer from postoperative severe anemia and hypoproteinemia often have a poor nutritional status and a poor quality of life, which further leads to low immunity, which may shorten the survival time and reduce overall survival for PGC patients. Therefore, this may be another important reason for better survival after PG-DTR.

A characteristic of traditional PG-DTR surgery is the dual output channel: one output channel is the jejunum-residual stomach-duodenal-jejunum, and the other output channel is the continuous jejunum, which can divide food. If tumor recurrence is observed in the surgical area or if one channel is obstructed, food can still pass through the other channel. Therefore, patients undergoing PG-DTR rarely require reoperation to relieve the obstruction. However, the output channel of the jejunum is an important section of the jejunum and naturally facilitates the passage of food. Therefore, if food passes through the continuous output channel too quickly, it is likely that little or no food will pass through the other output channel, which is the jejunum-remnant stomach-duodenum-jejunum. In this case, digestive juices such as gastric juice and pancreatic juice may not be secreted, which may contribute to the incomplete digestion of food and causes fullness, dyspepsia, loss of appetite, and even intestinal obstruction and life-threatening symptoms. We largely optimized these shortcomings. We used jejunum sutures between the gastrojejunal anastomosis and Braun side-to-side anastomosis to narrow one half of the intestine by two to three stitches so that part of the food passed through the stomach from the duodenum to the jejunum, which played a good regulating role. This is a good solution to the above shortcomings. In addition, we tied and blocked the input loop approximately 5–7 cm away from the esophagojejunostomy with no. 7 silk instead of cutting off the jejunum to reduce one anastomosis and to effectively avoid the possibility of postoperative peritonitis caused by leakage of jejunostomy. Moreover, PG-DTR is suitable for most patients undergoing digestive tract reconstructions after proximal gastrectomy, which has low requirements on the residual stomach, especially for patients with excessive gastrectomy who are unsuitable for esophagogastric residual anastomosis.

This study has several limitations. First, the surgical instruments evolved and the surgical level increased throughout the study trial, and the surgeons who performed the PGC surgery had less experience with PG-DTR than with TG, which could have affected clinical outcomes. Second, the screening data were incomplete, and thus selection bias could not be completely ruled out. Third, based on a systematic review of 34 randomized controlled studies [27], Peters found that compared with chemotherapy alone, postoperative chemotherapy for patients with gastric cancer had a significant beneficial effect on overall survival. Chang et al. [28]found that the long-term survival rate and the disease-free survival rate of postoperative patients with stage III gastric cancer receiving adjuvant chemotherapy were higher than of those receiving adjuvant chemotherapy alone. However, according to Drake et al.’s study [29], adjuvant chemotherapy after surgery in patients with gastric cancer was associated with slight improvement in overall survival, and it was not yet clear which patients benefit from it. Unfortunately, we did not include patients who received adjuvant chemotherapy after surgery in this study. Since the patient did not receive regular adjuvant chemotherapy in the hospital after surgery, the data were incomplete. We will expand the sample size to collect more reliable data in the future. Fourth, the choice of the two surgical procedures was based on the distance between the PGC distal tumor margin and the pylorus. Guidelines have suggested that the gastric incision line should be no less than 5 cm from the tumor margin [30, 31]. Therefore, we specified that the gastric incision line should be no less than 5 cm from the distal tumor margin. If the distance between the distal tumor margin and the pylorus was less than or equal to 5 cm, TG was used. PG-DTR was used when the distance between the distal tumor margin and the pylorus was greater than 5 cm. Finally, the median follow-up time was too short to provide relevant information on longer survival comparisons and chronic complications. Therefore, it is necessary to have continuous and long-term follow-up for patients with PGC.

In our study, we found that long-term oncologic outcomes were significantly higher and postoperative complication prevention was significantly more effective in patients with PGC who underwent PG-DTR than in those who underwent TG. Compared to TG, PG-DTR had longer 5-year overall survival and prevented postoperative complications, such as more severe anemia and hypoalbuminemia. Thus, our findings further suggest that PG-DTR may be a valuable and promising surgical procedure for patients with PGC.

## Conclusions

The patients who underwent PG-DTR had a favorable prognosis. Compared to TG, PG-DTR can alleviate postoperative complications, such as severe GR, anemia, and hypoalbuminemia. Thus, PG-DTR is more beneficial for patients with PGC and may be a valuable and promising surgical procedure.

## Data Availability

The datasets supporting the conclusions of this article are included within the article. The first author can be contacted to provide raw data if needed.
